# MicroRNA-876-5p inhibits the progression of glioblastoma multiforme by directly targeting Forkhead box M1

**DOI:** 10.3892/or.2022.8360

**Published:** 2022-07-04

**Authors:** Lina Wang, Jia Lu, Hong Zhang, Xueman Lyu, Zhigang Sun

Oncol Rep 41: 702–710, 2019; DOI: 10.3892/or.2018.6804

Following the publication of this paper, it was drawn to the Editors' attention by a concerned reader that the data shown in one of the panels belonging to the flow cytometric experiments shown in Fig. 5C were strikingly similar to data appearing in different form in another article published by different authors. Owing to the fact that the contentious data in the above article were already under consideration for publication prior to its submission to *Oncology Reports*, the Editor has decided that this paper should be retracted from the Journal. After having been in contact with the authors, they agreed with the decision to retract the paper. The Editor apologizes to the readership for any inconvenience caused.

